# Health-related medium term quality of life in intermediate risk pulmonary embolism in a general icu

**DOI:** 10.1186/2197-425X-3-S1-A768

**Published:** 2015-10-01

**Authors:** E Portugal Rodríguez, M Del Valle Ortiz, O Badallo Arévalo, JM Ayuela Azcárate, E Martínez Barrio, A Berrazueta Sánchez de Vega, M Gero Escapa, JA Fernández Ratero, SA Ossa Echeverri, ME Perea Rodríguez, C Carbajales Pérez

**Affiliations:** Burgos University Hospital, Burgos, Spain

## Introduction

Quality of life (QOL) after an episode of pulmonary embolism (PE) may be influenced by the factors related with the PE and its complications.

## Objectives

The aim is to determine whether poor clinical condition after discharge (6-12 months) of patients with intermediate-risk (IR) PE is influenced by epidemiological and clinical factors, echocardiography (TTE) on admission and/or discharge; analytical and electrocardiographic (ECG); specific treatment and associated complications[1].

## Methods

Descriptive, observational study of patients with IR PE admitted to the ICU during a 5 years period (2010-2014). IR PE was classified by PESI. QOL was analyzed 6-12 months after discharge, into two subgroups: good clinical condition (no dyspnea, normal life, partial dependence) or poor condition (dyspnea, total dependence). Analysis: Chi square and Fisher exact test. Variables: epidemiological (venous thrombosis, previous embolism, oral contraceptive, immobilization, surgery, smoking, neoplasia, heart and bronchial disease); clinics (syncope, chest pain, heart rate>110 bpm, Fi02>30%); analytical (troponin, proBNP, D-Dimer, pH, pC02); ECG; TTE (right cavities dilatation (RCD), TAPSE< 15 mm, tricuspid regurgitation (TR), pulmonary hypertension (PHT), Mc.Connell sign); treatment applied (fibrinolysis or anticoagulation only)[2] and complications (mechanical ventilation-MV, bleeding, home 02).

## Results

81 cases of PE; 67 cases IR were selected (56.7% male). Mean age: 66.31 ( ± 16.32) years. Two subgroups: 58.5% had good QOL while 41.5% had poor condition at 6-12 months from discharge. We related all variables with poor QOL obtaining: no statistically significant relationship (SSR) with epidemiological factors and ECG, correlation with clinical factors: acidosis on admission (p 0.001) and Fi02 (p 0.014) and hypercapnia (p 0.028) at discharge. TTE factors were analysed: TAPSE, RCD on admission and TR at discharge were SSR with poor QOL (p 0.001, p 0.039, p 0.034). There was no association with the treatment applied. We found worst QOL in those who needed MV (p 0.02) had bleeding complications (p 0.003) and required home 02 (p 0.016).

## Conclusions

A poor QOL after IR PE discharge (6-12 months), is related with acidosis, low TAPSE and RCD on admission; need of MV, high Fi02, bleeding and hypercapnia complications during ICU stay; TR and needs of home 02 at discharge.
